# Remembering for resilience: Brief cognitive‐reminiscence therapy improves psychological resources and mental well‐being in young adults

**DOI:** 10.1111/aphw.12364

**Published:** 2022-05-02

**Authors:** David John Hallford, Sarah Hardgrove, Meghna Sanam, Stefany Oliveira, Megan Pilon, Tyler Duran

**Affiliations:** ^1^ School of Psychology Deakin University 1 Gheringhap Road, Geelong Victoria Australia

**Keywords:** automatic thoughts, awareness of narrative identity, cognitive reappraisal, cognitive reminiscence therapy, meaning in life, optimism, reminiscence therapy, self‐efficacy, self‐esteem, young adults

## Abstract

Reminiscence‐based interventions focus on the recall of autobiographical memories and reflective reasoning about these remembered experiences. This study assessed the effect of a three‐session, positive‐memory version of cognitive‐reminiscence therapy (CRT) on the psychological resources and mental well‐being of young adults. The participants (*N* = 62, *M*_age_ = 24.6 [*SD* = 3.1], 71% females) were randomised to CRT or wait‐list. Psychological resources (self‐esteem, self‐efficacy, meaning in life and optimism), mental well‐being (depression, anxiety and stress symptoms) and theorised change processes (automatic negative thoughts, awareness of narrative identity and cognitive reappraisal) were assessed. The results showed the CRT group was significantly higher on psychological resources at post‐CRT (*d* = 0.75–0.80) and follow‐up (*d* = 0.52–0.87) and mental well‐being at post‐intervention (*d* = 0.71–1.30) and follow‐up (*d* = 0.64–0.98). The hypotheses regarding change processes were supported. Future research may use an active comparator and include a longer follow‐up, given only short‐term effects were assessed. Brief, positive‐focused CRT is effective in increasing psychological resources and mental well‐being in young adults.

## INTRODUCTION

Reminiscence‐based interventions involve the recall of autobiographical memories (i.e. memories of personal experiences) of specific events and periods across the lifespan. In reminiscence‐based interventions, these memories are reflected upon and considered for what can be learned about them and how they can be adaptively interpreted or integrated into one's sense of self. These reflections may be done independently, but often involve a guided review with the purpose of constructing a more rational, positive and meaningful understanding of oneself and one's life. Reminiscence‐based interventions can be thought of as a general approach through which other therapeutic modalities or techniques, such as cognitive‐behavioural, narrative or problem‐solving therapies can be delivered or integrated (Webster et al., 2010; Westerhof et al., 2010). Reminiscence‐based interventions have been shown to be effective across a diverse range of outcomes, such as meaning in life, self‐efficacy, depressive symptoms, positive well‐being and cognitive functioning (Pinquart & Forstmeier, 2012), and populations including depressive symptoms in clinically depressed individuals (Westerhof & Slatman, 2019), people with dementia (Park et al., 2019) and chronic physical disease (Pinquart & Forstmeier, 2012).

The vast majority of studies to date on reminiscence‐based interventions has involved older adult populations. This has been driven by the notion that perceiving one's life as having been coherent and meaningful is predominantly a psychosocial development task of older adulthood (Butler, 1963; Erikson, 1959). However, Hallford and Mellor (2013) have argued that young adults also have the developmental task of interpreting and evaluating their lives as coherent and meaningful and equally stand to benefit from reminiscence‐based approaches in achieving this. This is backed by evidence that young adults draw on autobiographical memory more frequently than older adults for the purposes of identity continuity and problem‐solving (Webster, 1993; Webster & McCall, 1999) that these adaptive uses of reminiscence predict decreases in depressive symptoms in young adults (Hallford et al., 2013; Hallford & Mellor, 2016a, 2016b, 2016c) and young adults report positive effects on self‐concept, affect and psychological resources such as self‐efficacy and meaning in life following brief reminiscence activities (Hallford & Mellor, 2016b; James & Bhar, 2016).

Based on this reasoning, Hallford and Mellor (2016a) evaluated a particular type of reminiscence‐based therapy, cognitive‐reminiscence therapy (CRT; Watt & Cappeliez, 2000), to treat help‐seeking young adults with clinically significant depressive symptoms. CRT involves a review of various life domains across six sessions (e.g. turning points, stressful experiences etc.), during which cognitive therapy (Beck et al., 1997) and stress and coping models and techniques (Billings & Moos, 1981) are incorporated to help challenge negative beliefs about the self and one's experiences and develop more realistic, positive and adaptive interpretations of life events. Theoretically, the aim is to recall and/or interpret experiences consistent with adaptive beliefs about the self, others and the world. This includes beliefs of oneself as being capable, of having intrinsic value and being valued by others, of having meaningful and cohesive experiences and as the world being a navigable place. These modified autobiographical memories are reconsolidated and theoretically integrated into adaptive global mental representations about the self, others and world (i.e. schema; e.g. I am worthwhile, I can cope with challenges) and act as evidence for them. Through this process, the improvement of psychological resources of self‐esteem, self‐efficacy, meaning in life and optimism are targeted as a means of alleviating depressive symptoms. While these are unlikely the only psychological resources that are affected through this process, they are consistent with the theorised processes of change (Watt & Cappeliez, 2000) that have been shown to mediate the associations between frequency of reminiscence and mental well‐being (Hallford et al., 2013; Hallford & Mellor, 2016a, 2016b, 2016c). A longer treatise on how this process is theorised to occur, and examples of such, can be found in Hallford and Mellor (2021). Consistent with prior research in older adults (Watt & Cappeliez, 2000), CRT caused large, significant reductions in depressive symptoms over time, as well as large increases in the targeted psychological resources. In addition to quantitative outcomes, interviews conducted with the young adults indicated that they found this autobiographical‐based intervention to be acceptable, appropriate and beneficial (Hallford et al., 2019).

In the present study we set out to extend upon this work in several ways. First, we aimed to replicate previous findings that CRT can have effects in this younger population and examine if it can enhance psychological resources in young adults that may not necessarily have elevated depressive symptoms. That is, can a reminiscence‐based intervention be beneficial for self‐esteem, self‐efficacy, meaning in life, and optimism in a community sample of young adults? These psychological resources are known contributors to overall psychological well‐being, and are in fact prophylactic against depressive symptoms (e.g. Hallford & Mellor, 2016b; Holden, 1992; Mascaro & Rosen, 2008; Paradise & Kernis, 2002; Scheier & Carver, 1985; Sowislo & Orth, 2013; Steca et al., 2014). Therefore, if CRT is effective in a community sample, then it would bolster confidence of this intervention approach in younger populations and demonstrate that it can enhance, rather than only remediate issues with, psychological resources. In theory, this approach is generalisable to non‐clinical samples as that the processes upon which it presumed to work (see above) are common to people across the spectrum of mental health or illness, and meta‐analyses indicate that it does improve positive well‐being among community samples not selected for health conditions (Pinquart & Forstmeier, 2012). It may then have utility for a range of therapeutic targets in subclinical or healthy youth populations, such as improving self‐esteem, confidence, or existential angst or anxiety. Given the target population was not those with clinical depression, it was determined that the sessions would focus primarily on positive past experiences rather than negative experiences and themes such as guilt, regret or shame, which are characteristics of depression (American Psychiatric Publishing, 2016). Positive past experiences that relate directly to the psychological resources of interest might be easily accessed in a community sample, for example, times of pride, success and meaning. Therefore, these were the focus of sessions rather than on redressing or reframing negative experiences. Further, as previous research shows that the majority of change in psychological resources occurs after only three sessions of CRT (Hallford & Mellor, 2016a), we opted to provide this number of sessions.

Second, we aimed to extend the understanding of variables that contribute to change in CRT. Young adults who engaged in CRT reported two key processes that, to the authors knowledge, have not yet been quantitatively studied in reminiscence‐based interventions (Hallford et al., 2019). The first was reappraising or changing their views of events and themselves. This was consistent with the facilitators goal of helping the young adults to understand and interpret their experiences in different ways and to challenge unhelpful beliefs or assumptions that might have arisen about themselves, their lives, others or the world. In the current study, we assessed whether participants reported changes in the frequency of negative thoughts about the self and the frequency with which they reappraised their thoughts. The second process identified by young adults was the increased awareness of a narrative or story about their lives. They reported that reviewing experiences from their life and linking these experiences together gave an overarching sense of continuity and meaning. This awareness meant that they could identify themes about their lives and themselves and comprehend a purpose for their actions and decisions. In the current study, we assessed participants' self‐reported awareness of a narrative identity, that is, perceiving life stories derived from past experiences that form part of one's identity.

Third, we aimed to evaluate this approach for young adults using an online, teleconference format. This format for talking‐based interventions provides significant advantages in terms of accessibility and convenience for participants and often less resources for providers. Most published evaluations of reminiscence‐based interventions have been of in‐person formats (Pinquart & Forstmeier, 2012),with some exceptions (Westerhof et al., 2019). Given the steady uptake of online mental health therapy and the strong evidence for its effectiveness in this format (e.g. Ahern et al., 2018; Berryhill et al., 2018), we reasoned it was appropriate to generate further evidence of online delivery of reminiscence‐based interventions.

The overall aim of this study was to examine the outcomes of a brief, reminiscence‐based intervention on the psychological resources of young adults and evaluate effects on other adaptive processes associated with change. It was hypothesised that the CRT group would have significantly higher self‐esteem, self‐efficacy, meaning in life and optimism than the control group immediately after the CRT sessions and at follow‐up. It was also hypothesised that the CRT group would report significantly lower frequency of negative automatic thoughts, greater use of cognitive reappraisal and a higher awareness of life stories. Although the participants were not recruited on the basis of poor mental well‐being, we reasoned that the sessions may have the effect of increasing mental well‐being, and therefore, it was hypothesised that the CRT group would report lower depressive, anxiety, and stress symptoms.

## METHODS

### Design

The study used a randomised controlled trial design with two arms (CRT; waitlist control) and three time‐points (baseline; post‐third session of CRT; 2‐week follow‐up). The primary outcomes were psychological resources of self‐esteem, self‐efficacy, meaning in life, and optimism. Secondary outcomes were processes of change, namely, negative automatic thoughts, use of cognitive reappraisal and awareness of narrative identity, as well as mental well‐being as assessed by depression, anxiety and stress symptoms.

### Participants

**FIGURE 1:**
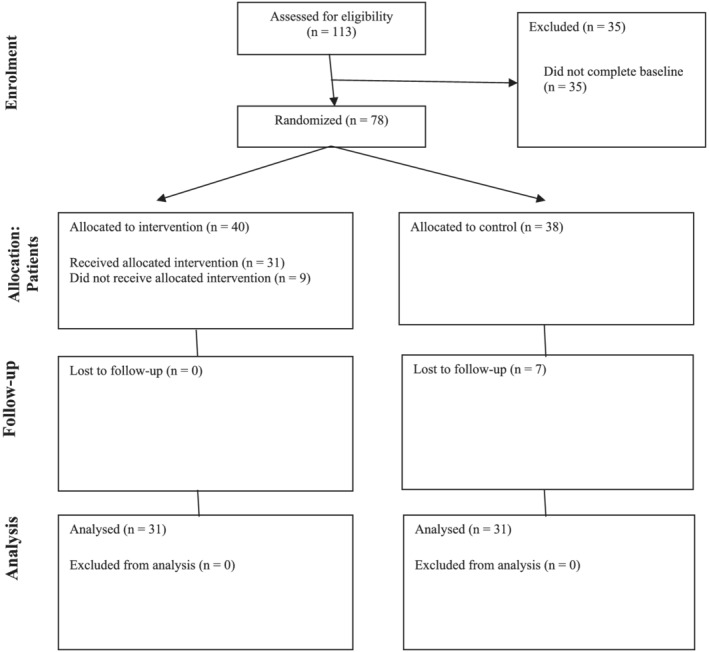
Participant flowchart

A community sample of young adults was recruited between June and September 2020 using advertisements distributed via social media and snowball sampling. Inclusion criteria were: aged between 18 to 29 years (representing emerging adulthood, Arnett et al., 2014), fluency in English, residing in Australia and internet access in the home. G*Power V3.1.9.4 (Faul et al., 2007) was used for an a priori power analysis. Previous effects of CRT on psychological resources in young adults have been large in magnitude (Cohen's *d* range 0.92–1.99; Hallford & Mellor, 2016a); however, this was observed in a sample with clinically significant depressive symptoms, which may have been more sensitive to positive change in psychological resources. We estimated a more conservative between‐groups effect size of *d* = 0.80; (1 − *β* = .80; α = .05), requiring a sample of at least 52 participants (26 in each condition) (Figure 1).

A total of 113 young adults registered interest in the study and 78 (40 CRT, 38 control) agreed to take part. Sixteen participants (nine CRT, seven control) withdrew from the study. Fourteen of these participants withdrew after baseline, and two withdrew after attending the first session. No differences were found between completers and non‐completers on age, *t*(77) = 0.51, *p* = .610; gender, χ^2^(1) = 0.02, *p* = .877; education level, χ^2^(1) = 0.03, *p* = .872; χ^2^(1). Ethnicity was approximately distributed across completers and non‐completers; however, chi‐square analysis could not be conducted due to violation of the minimum expected cell count frequencies.

A total of 62 participants (31 CRT; 31 control) completed the study and were included in the final analysis. As shown in Table 1, the average age was 24, over two‐thirds of the sample were female and had university‐level education, most were working, half were studying and the majority identified as Caucasian, followed by Asian, Latino and other ethnicities. As shown in Table 1, no differences were found between the groups on age or gender, while education and ethnicity were not analysed given small cell sizes.

**TABLE 1:** Characteristics of participants

Characteristic	Full sample	Control	CRT	Between group statistics
Age	24.68 (*SD* = 3.1)	24.84 (*SD* = 3.4)	24.52 (*SD* = 2.8)	*t* = 0.4, *p* = .691
Number of females	44 (71%)	21 (67.7%)	23 (74.2%)	χ^2^ = 1.1, *p* = .563
Level of education				
High school	21	10 (32.3%)	4 (12.9%)	
Diploma	7 (11.3%)	1 (3.2%)	6 (19.4%)	
Undergraduate degree	28 (45.2%)	11 (35.5%)	17 (54.8%)	
Postgraduate degree	13 (21%)	9 (29%)	4 (12.9%)	
Working	51 (82.3%)	26 (83.9%)	25 (80.6%)	χ^2^ = 0.1, *p* = .704
Studying	33 (53.2%)	14 (45.2%)	19 (61.3%)	χ^2^ = 1.6, *p* = .203
Ethnicity				
Caucasian/white European	41 (66.1%)	25 (80.6%)	16 (51.6%)	
Asian	13 (21%)	3 (9.7%)	10 (32.3%)	
Latino	4 (6.5%	1 (3.2%)	3 (9.7%)	
Other	4 (6.5%)	2 (6.5%)	2 (6.5%)	

*Note*: Ethnicity and level of education were not compared due to inadequate cell sizes for analyses.

### Materials

#### Self‐esteem

A shortened version of the Rosenberg Self‐Esteem Scale (Rosenberg, 2015) was used that included five positively worded items. This short‐form maintains the measurement fidelity of the full scale, with good convergent and divergent validity, and internal reliability (Hallford et al., 2013). Participants responded to items using an 11‐point, end‐defined self‐report scale ranging from 0 (do not agree at all) to 10 (agree completely). Responses were averaged together, with higher scores indicating higher self‐esteem. In the current study, internal reliability was found to be good (MacDonald's ω = .93).

#### Self‐efficacy

The eight‐item New General Self‐Efficacy Scale (NGSE; Chen et al., 2001) was used to assess perceived personal competence to deal effectively with a variety of stressful situations. Participants responded using a self‐report scale ranging from 1 (*strongly disagree*) to 5 (*strongly agree*). The responses to the items were averaged together, with higher scores on this scale indicating higher general self‐efficacy. The NGSE has demonstrated good psychometrics (Chen et al., 2001; Scherbaum et al., 2006). In the current study, internal reliability was found to be good (MacDonald's ω = .82).

#### Meaning in life

The five‐item Presence subscale of the Meaning in Life Questionnaire (Steger et al., 2006) was used to assess the extent to which participants felt that their lives were meaningful. This questionnaire has good psychometric properties and measures meaning in life as a distinct psychological construct (Steger et al., 2006). Participants used a self‐report scale ranging from 1 (*absolutely untrue*) to 7 (*absolutely true*). The item response scores were averaged together, with higher scores reflecting a stronger sense of personal meaning in life. In the current study, internal reliability was found to be good (MacDonald's ω = .81).

#### Optimism

A three‐item, short‐form version of The Life Orientation Test – Revised (LOT‐R; Carver et al., 2010) was used to measure optimism. This consisted of the positive worded items from the LOT, excluding three items that measure pessimism and four “filler” items. This short‐form has demonstrated good convergent and divergent validity and good internal reliability (α = .86; Hallford et al., 2013). Participants responded to items using a self‐report scale ranging from 1 (*I disagree a lot*) to 5 (*I agree a lot*). The responses were averaged together, with higher scores indicating more positive generalised outcome expectancies for the future. In the current study, internal reliability was acceptable (MacDonald's ω = .73).

#### Negative automatic thoughts

The eight‐item Automatic Thoughts Questionnaire (ATQ; Netemeyer et al., 2002) was used to assess how frequently various types of negative thoughts about oneself “popped” into people's heads over the last week, for example, “I'm no good” or “I'm so disappointed in myself.” The participants responded to items using a self‐report scale ranging from 1 (*not at all*) to 5 (*all the time*). This version of the ATQ has adequate psychometric properties relative to the longer 15 and 30‐item version (Netemeyer et al., 2002). In the current study, internal reliability was acceptable (MacDonald's ω = .83).

#### Cognitive reappraisal

The six‐item cognitive reappraisal subscale from the Emotion Regulation Questionnaire (Gross & John, 2003) was used to assess how strongly people agree that they cognitively change the meaning of experiences in order to regulate their emotions. The participants responded to items using a self‐report scale ranging from 1 (*strongly disagree*) to 7 (*strongly agree*). In the current study, internal reliability was good (MacDonald's ω = .91).

#### Awareness of narrative identity

The five‐item awareness subscale of the Awareness of Narrative Identity Questionnaire (Hallford & Mellor, 2017) was used to assess how strongly people perceived that there were stories about their life that could be derived from their past experiences (e.g. “when I think over my lifetime, I can observe how there is a story that tells me who I am”). This subscale has demonstrated good psychometric properties and construct validity by correlating with various indices of the coherence of written narratives about people's lives (Hallford & Mellor, 2017). Participants responded to items using an 11‐point, end‐defined self‐report scale ranging from 0 (*do not agree at all*) to 10 (*agree completely*). In the current study, internal reliability was good (MacDonald's ω = .95).

#### Depression, anxiety and stress symptoms

The 21‐item version of the Depression, Anxiety and Stress Scale (DASS‐21; (Lovibond & Lovibond, 1995) was used to assess mental well‐being via core symptoms of depression, anxiety and stress. Each subscale consists of seven self‐report items that are each rated on a 4‐point self‐report scale from 0 (*did not apply to me at all*) to 3 (*applied to me very much*, or *most of the time*). The subscales possess very good psychometric properties (Antony et al., 1998) and have been validated for use with young adults (Mahmoud et al., 2003). In the current study, internal reliability was acceptable for all subscales (MacDonald's ω: depression = .85, anxiety = .80, stress = .81).

### Cognitive‐reminiscence therapy protocol

This three‐session version of CRT was focused on positive experiences and adapted from Hallford and Mellor (2016a, 2016b, 2016c) version of CRT. It is manualised for standardised delivery and available with the full six session manual for clinical depression (please contact the first author for a copy). The program comprised three, weekly 60‐ to 90‐min sessions centred on three consecutive topics: positive relationships, coping and overcoming and meaning in life. These topics were chosen based on the first author's experience in research and practice with CRT as they would cover some core areas of significance to young adults. Participants were asked to complete homework sheets before each session to facilitate memory recall and stimulate reflective processes to be used in the sessions. The questions asked participants to describe details of specific memories, related thoughts and feelings and subsequent impacts on their life. In the first session, facilitators established rapport and explained the session format, including group expectations. The facilitators provided psychoeducation on reminiscence and its functions. They drew on examples to illustrate what was sought regarding level of detail, surrounding context and accompanying thoughts and feelings. The facilitators emphasised the value of sharing aspects of memories relating to attitudes towards oneself and one's abilities, personal growth and significance of experiences in the context of one's life. To identify suitable memories, participants took turns to provide a summary of memories they wished to share. As participants shared their memories, the facilitators asked questions to encourage elaboration of details (e.g. “do you remember the sights and sounds?”), to identify thoughts and feelings (e.g. “can you remember what you were thinking and feeling at the time?”) and uncover unexpressed meaning (e.g. “what was most important about that experience for you?” and “how might this experience have impacted your life in other ways?”). Specific questions were asked with the aim of using reflective reasoning to increase the perception of psychological resources. For example, participants were asked how positive events made them feel about themselves, to identify specific aspects of successful coping and adaptation as well as the outcomes of this, to describe experiences that were personally meaningful and why, and to think about how a positive experience (e.g. indicating personal worth, self‐efficacy or meaning) might affect how they think about their future and their future self. Each participant shared one or two memories per session. Following the individual sharing, time was provided for group reflection on their experience of the reminiscence activity, and any new thoughts or feelings that might have emerged (e.g. “did thinking about your past experiences give you any new personal insights?”). Before closing the session, participants were reminded of the value of completing homework as a tool to prime memory recall and amplify the potential for increases in psychological resources. Extensive elaboration on these processes can be found in the manuals and in Hallford and Mellor (Hallford & Mellor, 2021).

### Procedures

Prior to recruitment, the study received ethical approval from the Deakin University Human Ethics Advisory Group (Approval ID: HEAG‐H 82_2020). Participants registered interest through online advertisements. They were then assessed for eligibility, and after providing informed consent, participants were randomised into a condition using a computer‐based algorithm (www.randomizer.org) with a 1:1 ratio. Participants in the CRT condition were emailed homework sheets to complete before each session. Starting approximately 1 week after baseline, participants engaged in group sessions delivered over 3 weeks using a teleconference platform. The groups ranged in size from 3 to 5. One participant was unable to attend one of the sessions, and two participants received individual sessions to make up for missed group sessions. The sessions were primarily facilitated by five students as part of completing their thesis for their fourth‐year of undergraduate psychological studies. The first author provided training and supervision on providing CRT. Regarding fidelity, the facilitators followed a simple checklist indicating the tasks they were to complete in each session (outlined above). Fidelity was good during sessions that were observed by the first author, and fidelity was checked for in supervision sessions to ensure that facilitators delivered the prescribed intervention.

Participants were emailed links to complete the study measures after the third session, and again 2 weeks later. The measures was estimated as taking approximately 30 min to complete. Once the study was completed, participants in the wait‐list condition were offered the opportunity to engage in the same CRT program. All participants who completed the study were offered a $10 retail voucher.

### Data analysis plan

IBM SPSS Statistics Version 27 was used for all statistical analyses. Descriptive statistics were generated for variables at each time point. Pearson correlations were computed to examine the zero‐order association of variables at baseline. MacDonald's ω is used above to assess the internal reliability of the multi‐item measurement scales, having superior properties and performance than Cronbach's alpha such as not (erroneously) assuming that each item measures the latent variable with equivalent precision (see Hayes & Coutts, 2020). To test the main hypotheses, analysis of covariance (ANCOVA) tests were used to obtain a valid estimation of the intervention effect, defined by differences between the groups at post‐session and follow‐up while adjusting for the baseline scores ((Frison & Pocock, 1992; Twisk & Proper, 2004; van Breukelen, 2013; Vickers & Altman, 2001). Estimation of the intervention effect through means and standard errors adjusted for baseline scores reflected the group by time interaction. To control for Type 1 errors, with α set at .05, the results from the ANCOVA analyses were subject to the false discovery rate procedure (Benjamini, 2010). Relative to multiple comparison corrections that control the familywise error rate to avoid making a single type I error, this procedure provides more statistical power to identify effects by aiming to control only the proportion of significant results that are Type I errors. A corrected significance level is provided after the procedure (*q*). Standardised mean difference score, Cohen's *d*, was used as a measure of effect size and interpretation followed Cohen's (Cohen, 1992) guidelines (small = .2, medium = .5, large = .8). The data that support the findings of this study are available on request from the corresponding author. The data are not publicly available due to privacy or ethical restrictions.

## RESULTS

The baseline correlations between the variables are reported in Table 2 while the descriptive statistics and results of inferential tests are reported in Table 3. All study variables were generally significantly correlated with one another, from small‐to‐moderate to large magnitudes. The exception was the awareness of narrative identity which had no significant correlations with other variables but did have non‐trivial (but non‐significant) associations with meaning in life and cognitive reappraisal. A series of *t*‐tests showed there were no significant differences between outcome variables at baseline (all *t*'s < 1.74, all *p*'s > .087).

**TABLE 2:** Correlations between study variables at baseline

	Esteem	Efficacy	Meaning	Optimism	ATQ	Reappraisal	ANIQ	DASS‐D	DASS‐A	DASS‐S
Esteem	‐									
Efficacy	.63***	‐								
Meaning	.59***	.31*	‐							
Optimism	.61***	.60***	.45***	‐						
ATQ	.59***	−.47***	−.51***	−.44***	‐					
Reappraisal	.58***	.44***	.42***	.51***	−.41***	‐				
ANIQ	.11	.06	.23	.08	−.07	.15	‐			
DASS‐D	−.51***	−.46***	−.45***	−.54***	.62***	−.48***	−.10	‐		
DASS‐A	−.25*	−.37**	−.10	−.28*	.42***	−.28*	.00	.36**	‐	
DASS‐S	−.24	−.29*	−.19	−.30*	.29*	−.40**	.01	.51***	.57***	‐

* *p* < .05,

** *p* < .01,

*** *p* < .001.

**TABLE 3:** Comparison of outcomes between control and CRT groups across time

Outcomes	Control group	CRT group	Adjusted difference (CRT ‐ control)		Corrected *p‐*value (*q*)
	Mean (*SD*)	Mean (*SD*)	Mean (95% CI)	Cohen's *d*	
Self‐esteem					
Baseline	7.24 (1.57)	7.01 (1.46)			
Post‐CRT	7.16 (2.08)	7.84 (1.32)	0.87 (1.47, 0.27)	0.75	.007
Follow‐up	7.50 (1.39)	7.80 (1.52)	0.46 (0.92, 0.01)	0.52	.045
Self‐efficacy					
Baseline	3.80 (0.64)	3.83 (0.46)			
Post‐CRT	3.66 (0.88)	4.12 (0.45)	0.44 (0.73, 0.15)	0.80	.005
Follow‐up	3.76 (0.81)	4.19 (0.46)	0.41 (0.65, 0.16)	0.87	.003
Meaning in life					
Baseline	4.70 (1.01)	5.02 (1.12)			
Post‐CRT	4.74 (1.35)	5.60 (0.94)	0.60 (0.99, 0.21)	0.80	.005
Follow‐up	4.97 (1.17)	5.60 (0.93)	0.40 (0.78, 0.02)	0.55	.042
Optimism					
Baseline	3.53 (0.95)	3.64 (0.74)			
Post‐CRT	3.57 (0.99)	4.13 (0.60)	0.48 (0.79, 0.17)	0.80	.005
Follow‐up	3.76 (0.77)	4.10 (0.64)	0.28 (0.52, 0.03)	0.58	.030
Automatic thoughts					
Baseline	2.09 (0.73)	1.94 (0.44)			
Post‐CRT	1.86 (0.72)	1.45 (0.30)	−0.35 (−0.52, −0.10)	0.74	.006
Follow‐up	1.87 (0.68)	1.42 (0.30)	−0.38 (−0.62, −0.14)	0.85	.005
ANIQ awareness					
Baseline	6.81 (1.99)	7.58 (1.63)			
Post‐CRT	6.30 (2.11)	8.12 (1.51)	1.82 (2.77, 0.87)	0.99	.003
Follow‐up	6.82 (1.83)	8.15 (1.44)	1.31 (2.16, 0.46)	0.79	.005
Emotion regulation ‐ reappraisal					
Baseline	4.43 (1.10)	4.83 (1.01)			
Post‐CRT	4.27 (1.10)	5.24 (0.80)	0.68 (1.00, 0.37)	1.00	.003
Follow‐up	4.39 (1.11)	5.20 (1.11)	0.53 (0.96, 0.10)	0.64	.018
DASS depression					
Baseline	4.56 (4.26)	3.70 (3.10)			
Post‐CRT	4.75 (3.73)	2.08 (2.06)	−2.22 (−3.40, −1.06)	1.30	.003
Follow‐up	4.82 (4.31)	1.83 (1.62)	−2.57 (−3.97, −1.18)	0.95	.003
DASS anxiety					
Baseline	3.73 (3.50)	2.52 (1.66)			
Post‐CRT	3.13 (3.06)	1.14 (1.36)	−1.42 (−2.46, −0.37)	0.71	.012
Follow‐up	2.35 (2.16)	0.62 (0.67)	−1.44 (−2.22, −0.67)	0.98	.003
DASS stress					
Baseline	6.69 (3.69)	5.71 (3.18)			
Post‐CRT	6.02 (4.25)	3.43 (2.37)	−2.01 (−3.45, −0.57)	0.73	.010
Follow‐up	5.65 (3.40)	3.50 (2.68)	−1.75 (−3.16, −0.34)	0.64	.018

*Note*: The adjusted difference reflects the group × time interaction.

The results of the ANCOVA analyses, which adjusted for baseline scores, showed the CRT group was significantly higher in all four measures of psychological resources than the control group after the last session of CRT and at the 2‐week follow‐up. The effects were moderate in size for self‐esteem, meaning in life and optimism and large for self‐efficacy at follow‐up. The findings for the change processes were largely the same, with significantly higher scores in the CRT compared with the control group after the last session of CRT and at the 2‐week follow‐up. The effects for change processes of negative automatic thoughts, awareness of narrative identity and cognitive reappraisal were of a moderate to large magnitude. For depression, anxiety and stress symptoms, the CRT group reported significantly lower scores compared with the control group after the last session of CRT and at the follow‐up, with large effects for depressive and anxiety symptoms and moderate to large for stress at the follow‐up.

## DISCUSSION

This study aimed to examine whether a brief CRT protocol, focused on positive memories and conducted online in a teleconference format, would improve psychological resources in young adults relative to a control group. Further, it aimed to examine whether CRT would have effects on hypothesised change processes and on mental well‐being as assessed by depression, anxiety and stress symptoms.

The hypothesis that psychological resources would be improved by CRT, relative to a control group, was supported with significantly higher self‐esteem, self‐efficacy, meaning in life and optimism reported at post‐CRT and follow‐up. The effects were generally moderate by the 2‐week follow‐up, although the effect on self‐efficacy remained large. These findings are consistent with previous studies of CRT in young adults with clinically‐significant depressive symptoms (Hallford & Mellor, 2016a, 2016b, 2016c) and extend on this by showing that a brief form of this intervention is effective for psychological resources in community samples of young adults. CRT also improved mental well‐being as assessed by depression, anxiety and stress scores. The magnitude of these effects were somewhat surprising given the sample had, on average, only mildly elevated scores on these indices relative to adults in the Australian general population (Crawford et al., 2011). However, this may be due to the reduction in the standard deviation between baseline and subsequent time‐points. This relatively smaller dispersion of scores around the mean would have been a factor in increased effect size using Cohen's *d*. Regardless, the results indicate that brief CRT can be effective for emerging adults in reducing mildly‐elevated symptoms of depression, anxiety, and stress.

This study is one of few to date that have specifically tested a reminiscence‐based intervention approach with young adults. Further, it provides support for the effectiveness of reminiscence‐based therapies in an online, teleconference group format. Although it is not clear if an in‐person approach would be more effective, the size of the observed effects in this study and the high accessibility and low resource burden of the online format has significant merit unto itself. Unfortunately, no evaluation data was collected, however, previous interview‐based research has indicated that this approach is viewed as acceptable for young adults (Hallford et al., 2019).

The findings regarding automatic negative thoughts, awareness of narrative identity, and cognitive reappraisal further our knowledge of the processes of change in CRT. Congruent with our rationale, the tendency to experience automatic negative thoughts about oneself reduced after CRT. This is consistent with the aim of CRT to explore various emotionally valenced experiences in order to balance appraisals of one's self‐concept. In particular, in this brief version there was a focus on the positive aspects of experiences and how this related to one's self‐worth. The awareness of narrative identity also increased following CRT. This was indicated by young adults in a previous study (Hallford et al., 2019), but assessed quantitatively here for the first time. This awareness may have occurred by participants being prompted to consider the overall significance and meaning of the experiences that were discussed in relation to one's identity, as well as whether they identified any themes about their lives that emerged. Listening to other participants' discussions of their personal experience may have also triggered other memories or an abstraction of memories increasing this awareness of “stories” about one's life. This process of identification is common to group interventions, and possibly a factor in other effects in this study too. Cognitive reappraisal was also found to increase, which is consistent with how people were asked to reflect on how they felt at the time of experiences, how they might think about them, how they might think about them differently, and how this might affect how they subsequently emotionally felt about them. In a sense, this constitutes implicit training in the effect of changing one's thinking to regulate their emotion. Of course, all three of these “change” processes could be considered to be positive outcomes themselves, but were conceptualised as change processes given, they were identified as such by young adults (Hallford et al., 2019).

There were several limitations to the study and its generalisability. Without an active control group, it is difficult to untangle specific CRT processes from other, common processes in empathic and supportive group settings. Future research could use a “simple reminiscence” intervention involving free‐form discussion about past experiences. This would help assess whether specific techniques within CRT produce stronger effects on psychological resources and well‐being, relative to general conversation about positive experiences. Although guided, theoretically‐driven reminiscence interventions have been shown to produce stronger effects than these “simple reminiscence” interventions (Pinquart & Forstmeier, 2012), a direct empirical test of this with CRT is needed. Adopting outcome measures such as life satisfaction in future would also allow for the assessment of effects on how young people more generally perceive their lives, especially given that such effects are observed in samples of older adults (Tam et al., 2021). Other measures could include specific forms of resilience in the face of adversity. A longer follow‐up is also needed to assess longer term effects than the 2‐week, short‐term effects assessed here. Maintenance of effects might be predicted given previous findings that effects last at least 3 months among youth with depression (Hallford & Mellor, 2016a, 2016b, 2016c), and more generally are maintained in reminiscence therapies at follow‐ups (Pinquart & Forstmeier, 2012). It would also be interesting to see if CRT had prophylactic effects on the development of clinical depression, given it did reduce depressive symptoms, and these psychological resources are themselves protective against future depressive symptoms. Correspondingly, further information on the sample characteristics, such as how many people may have had clinical disorders, would have aided somewhat in describing the sample. The study relied solely on self‐report which, although appropriate for assessing personal knowledge such as emotions and self‐concept, might be supplanted in future by behavioural measures (such as amount or duration of engagement in meaningful activities) or observational outcomes by third parties such as friends or family.

It is noteworthy that this study was conducted during the COVID‐19 pandemic, and particularly at a time of widespread restrictions in Australia. An intervention such as CRT that focuses on reviewing past events rather than goal‐directed behavioural change might have been highly appropriate during a time of restrictions on possibilities for socialising, meaningful activities and possibly demoralisation and an increased sense of isolation and hopelessness. The outcomes of the study do suggest that a period of time such as this, during which people may benefit greatly from increased resilience, an intervention that uses retrospection might be useful and appropriate. In conclusion, this brief, positive‐focused, teleconference‐delivered version of cognitive‐reminiscence therapy was effective in increasing psychological resources and mental well‐being in young adults.

## ETHICS STATEMENT

This study was approved by the Deakin University Health Ethics Advisory Group. ID: HEAG‐H 82_2020.

## CONFLICT OF INTEREST

The authors have no conflicts of interest to declare.

## Data Availability

The data that support the findings of this study are available on request from the corresponding author. The data are not publicly available due to privacy or ethical restrictions.
